# Acute Distal Vertebral Artery Occlusion in Patients with Asymmetrical Vertebral Artery Geometry: Role of Black-Blood-Enhanced MR Imaging

**DOI:** 10.3390/diagnostics12102391

**Published:** 2022-10-01

**Authors:** Youngsun Jeong, Chan Sol Park, Ui Yun Lee, Seung Bae Hwang, Gyung Ho Chung, Hyo Sung Kwak

**Affiliations:** 1 Jeonbuk National University Medical School, Jeonju 54907, Korea; 2 Division of Mechanical Design Engineering, College of Engineering, Jeonbuk National University, Jeonju 54896, Korea; 3 Department of Radiology and Research Institute of Clinical Medicine of Jeonbuk National University, Biomedical Research Institute of Jeonbuk National University Hospital, Jeonju 54907, Korea

**Keywords:** stroke, magnetic resonance imaging, black blood imaging, contrast enhancement

## Abstract

Background: The purpose of this study was to evaluate the diagnostic value of contrast enhancement in a unilateral distal vertebral artery (VA) using black blood (BB)-enhanced magnetic resonance (MR) imaging in patients with acute neurological symptoms and asymmetrical VA geometry. Methods: From January 2020 to August 2021, we retrospectively analyzed BB-contrast-enhanced MR imaging and MR angiography (MRA) findings in stroke patients visiting the emergency room for an evaluation of acute neurological symptoms. We classified four patterns according to asymmetrical VA geometry using MRA and contrast enhancement using BB-enhanced MR imaging: type 1 = enhanced VA + no visualization of VA, type 2 = enhanced VA + hypoplastic VA, type 3 = non-enhanced VA + hypoplastic VA, or type 4 = non-enhanced VA + no visualization of VA. Results: In total, 288 patients (type 1 = 65, type 2 = 17, type 3 = 130, type 4 = 76) were enrolled in this study. Of these patients, 82 (28.5%) showed contrast enhancement of a unilateral distal VA on BB-enhanced MR imaging, and 51 (17.8%) had positive findings on diffusion-weighted imaging (DWI) in the ipsilateral medulla, pons, or posterior inferior cerebellar artery (PICA) territory. The contrast enhancement of a unilateral distal VA using BB-enhanced MR imaging demonstrated a significantly higher prevalence in patients with acute infarction on DWI (50.0% vs. 4.9%, *p* < 0.001). Conclusions: The contrast enhancement of a unilateral distal VA on BB-enhanced MR imaging is associated with acute infarction of the medulla, pons, or PICA territory and suggests acute occlusion of a distal VA.

## 1. Introduction

Stroke involving the vertebrobasilar system represents approximately 20–25% of all ischemic strokes [1]. Steno-occlusion of a vertebral artery (VA) accounts for approximately 20% of posterior circulation ischemia [2]. If the blood supply to the VA is sufficiently reduced, neurologic symptoms of vertebrobasilar arterial (VBA) ischemia may occur [3]. Therefore, early detection of arterial occlusion comes with invaluable prognostic and therapeutic outcomes for patients with ischemic events [4].

Various imaging modalities, such as CT or stroke MR protocol, including perfusion imaging, are used to evaluate an arterial occlusion site and restore cerebral function. In general, in stroke MR protocol, angiography and susceptibility vessel sign (SVS) on susceptibility-weighted imaging (SWI) show an occlusion site of an intracranial artery and a thrombus within the occluded site [5,6]. Additionally, an SVS in the posterior circulation is significantly superior to non-contrast CT for thrombus detection [6,7].

Black blood (BB) magnetic resonance (MR) imaging, a technique that selectively suppresses signals from flowing arterial blood, is used to visualize and quantify the size of an arterial lumen and the outer wall boundaries of large arteries. A strong enhancement of the intracranial artery in BB-enhanced MR imaging was especially seen in patients with thrombotic occlusion or severe stenosis [8,9,10]. Additionally, BB-enhanced MR imaging had a higher sensitivity than that of SVS on SWI for the detection of an intra-arterial thrombus in patients with acute stroke of the posterior circulation [11]. However, there are no studies associated with BB-enhanced MR imaging for detecting acute infarction related to the occlusion of a distal VA. We hypothesized that the acute occlusion of a unilateral VA was associated with infarction in the medulla, pons, or posterior inferior cerebellar artery (PICA) territory, and BB-enhanced MR imaging can detect an acute occlusion of a unilateral distal VA. The purpose of this study was to evaluate contrast enhancement patterns on BB-enhanced MR imaging and MR angiographic patterns in patients with acute neurological symptoms and asymmetrical VA geometry.

## 2. Materials and Methods

Our institutional review board approved the retrospective study protocol, and the requirement for informed consent was waived for the review of patient medical records and images (JUH2021-09-008, Sep-2021).

### 2.1. Patients

From January 2020 to August 2021, we retrospectively reviewed stroke MR examinations with BB-enhanced MR imaging and MR angiography of patients visiting the emergency room for evaluation of acute neurological symptoms. Exclusion criteria were as follows: (a) MR examination 48 h after symptom onset; (b) evidence of vertebral dissection or vasculitis; (c) slow flowing artifact or incomplete suppression of VA lumen in BB-enhanced imaging; (d) any patient with insufficient quality for reliable evaluation in BB-enhanced MR imaging or MR angiography; (e) MR imaging after endovascular treatment due to acute occlusion of posterior circulation; (f) evidence of symptomatic stenosis of basilar artery (BA); or (g) mismatched lesion between unilateral enhancement of VA and positive lesion on diffusion-weighted imaging (DWI).

### 2.2. MRI Procedure

MR examination of this study was performed using an Achieva 3.0-T MRI scanner (Philips Healthcare, Best, The Netherlands) with a 16-channel head coil. MRI for ischemic symptom evaluation was undertaken after an initially non-enhanced brain CT scan, which was conducted to rule out intracranial hemorrhage. Our MR protocol included the following sequences: (a) DWI; (b) 3D time-of-flight (TOF) MRA of the intracranial arteries; (c) susceptibility-weighted imaging (SWI); (d) BB-enhanced MR imaging of whole brain; (e) contrast-enhanced carotid MR angiography; and (f) FLAIR imaging.

The protocol was previously described [8,11,12]. BB-enhanced MR imaging was undertaken using volumetric isotropic turbo spin-echo acquisition (VISTA; Philips Healthcare) in the coronal plane (slab thickness = 40 mm) for flow suppression. We used the improved motion-sensitized driven-equilibrium (iMSDE) method, which suppresses enhanced signals in blood vessels [13,14]. Acquisition parameters for iMSDE–VISTA images were as follows: repetition time/echo time = 450.0/22.4 ms, flip angle = 90°, echo train = 26, sensitivity encoding = 2, field of view = 256 × 256 mm, matrix = 256 × 256, 1 mm slice thickness and no gap, and scan time = 35–38 s. Gadodiamide (0.1 mmol/kg body weight; Dotarem; Guerbet, Aulnay-sous-Bois, France) was intravenously injected as a bolus in all patients. BB contrast-enhanced 3D MR imaging was carried out approximately 5 min after contrast injection. After image acquisition in the sagittal plane, we reconstructed images in the axial and coronal planes.

### 2.3. Clinical Data Analysis

Clinical data of the included patients, including basic demographics and risk factors for atherosclerosis, namely diabetes, hypertension, dyslipidemia, current smoking, and history of coronary disease, were recorded.

### 2.4. MR Imaging Analysis

All MR imaging, including BB-enhanced MR and MRA, was retrospectively reviewed by two neuroradiologists (with 30 years and 16 years of experience, respectively) blinded to the clinical information of each patient and the purpose of the study. Two neuroradiologists analyzed imaging quality, flow-related artifacts, presence of contrast enhancement, geometry of distal VA, and location of acute infarction on DWI. 

They assessed image quality by consensus. Poor imaging quality was defined as ill-defined visualization of VA or impossible analysis due to motion artifacts or incomplete coverage. Patients with poor image quality were excluded from the final analysis. Patients were analyzed according to whether TOF or contrasted-enhanced MRA findings of both VA suggested occlusion, hypoplasia, or normal. Distal VA hypoplasia was defined as a diameter with a difference of more than 2 mm [15]. 

Contrast enhancement of the VA lumen on BB-enhanced MR imaging was classified as follows: complete unilateral contrast enhancement of vessel lumen versus no contrast enhancement. Contrast enhancement was defined as a similar density compared to the enhanced pituitary gland and complete filling of the vessel lumen. The target appearance with peripheral or partial contrast enhancement and central black blood lumen was defined as no contrast enhancement due to different flow velocities and/or a slow flow effect. The target appearance was excluded from this study. 

**Figure 1:**
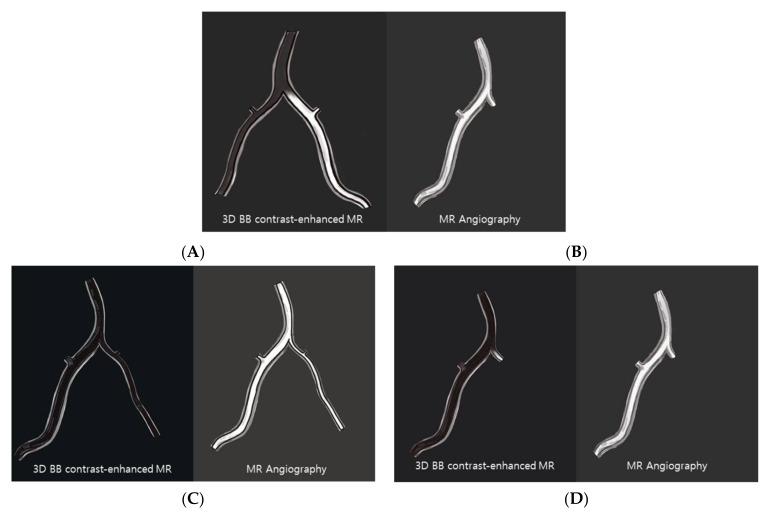
Four types of asymmetrical VA geometry on black blood enhanced MR and MRA. (**A**) Type 1 = unilateral contrast-enhanced VA + no visualization of VA on MRA; (**B**) Type 2 = unilateral enhanced VA + hypoplastic VA; (**C**) Type 3 = no enhanced VA + hypoplastic VA; (**D**) Type 4 = unilateral complete occlusion of VA + no visualization of VA.

We classified images using four types according to contrast enhancement of the unilateral distal VA on BB-enhanced MR imaging and asymmetrical VA geometry on MR angiography (Figure 1): 1 = unilateral contrast-enhanced VA + no visualization of VA on MRA; 2 = unilateral enhanced VA + hypoplastic VA; 3 = no enhanced VA + hypoplastic VA; and 4 = unilateral complete occlusion of VA + no visualization of VA. Type 1 was defined as contrast stagnation of the VA by an acute occlusion of the distal VA.

Medulla, pons, or PICA infarctions related to distal VA occlusion were defined as the detection of a hyperintense signal on a DWI trace with an associated signal decrease in the apparent diffusion coefficient map.

### 2.5. Statistical Analyses

All data analyses were processed using SPSS 24.0 software (SPSS, Chicago, IL, USA). Quantitative variables were expressed as a mean ± standard deviation or median, and categorical variables were expressed as frequencies and percentages. We performed a univariate comparison of groups using an independent *t*-test for continuous variables and a Pearson chi-square test for categorical variables.

## 3. Results

During this study period, 316 patients with asymmetrical VA geometry and BB-enhanced MR imaging were enrolled. Of these patients, 11 were excluded from this study due to MR imaging after endovascular treatment, 3 due to target appearance in enhanced imaging because of incomplete vessel suppression by slow flow, 2 due to VA dissection, and 2 due to poor imaging quality. Finally, 288 patients (167 males, mean age 71.4 years) were enrolled in this study. The demographic data of these patients are described in Table 1. 

**Figure 2:**
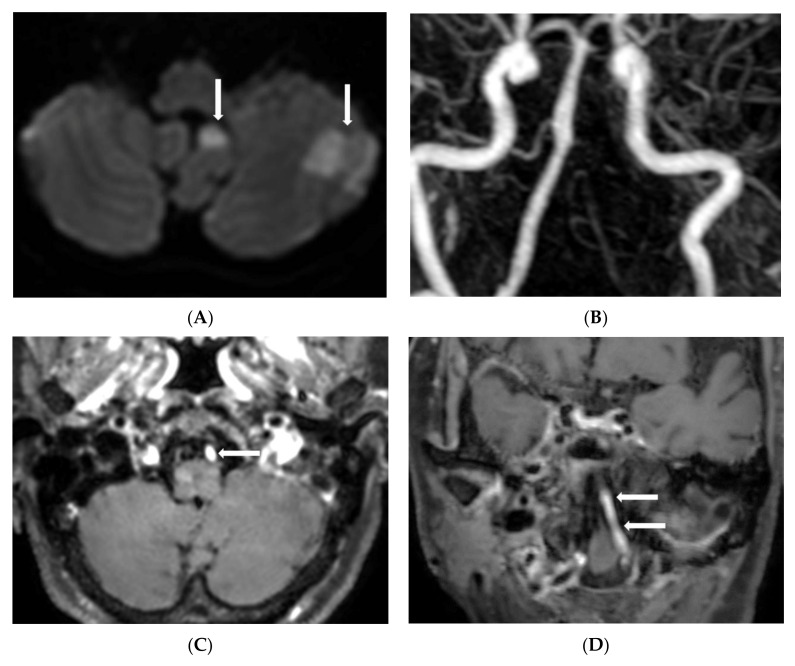
An 83-year-old woman with acute vertigo attack. (**A**) Diffusion-weighted imaging shows the acute infarction in the left posterior inferior cerebellar artery territory (arrows); (**B**) Contrast-enhanced MRA shows no visualization of the left vertebral artery; (**C**) Axial imaging of black-blood enhanced MR imaging shows strong enhancement of left vertebral artery (arrow); (**D**) Coronal reformatted imaging of black-blood-enhanced MR imaging shows long segment contrast enhancement of left vertebral artery (arrows). This finding suggests contrast stagnation by acute occlusion of the distal vertebral artery related to acute infarction of the posterior inferior cerebellar artery territory.

Of these patients, 129 had a type 3 pattern (non-enhanced VA + hypoplastic VA) and 77 had a type 4 (non-enhanced VA + no visualization of VA). Furthermore, 82 (28.5%) showed a contrast enhancement in a unilateral distal VA on BB contrast-enhanced MR imaging (type 1 = 65 and type 2 = 17). Additionally, 51 (17.8%) showed matched positive findings on DWI related to asymmetrical VA geometry. Of 51 patients, 22 had medulla infarction, 9 pons infarction, and 20 PICA territory infarctions (Figure 2).

A comparison of the two groups with enhanced VA or non-enhanced VA on BB MR imaging is shown in Table 2. Of 82 patients with a contrast enhancement of unilateral distal VA (type 1 and 2), 41 (50.0%) had matched positive findings related to territorial VA (medulla = 19, pons = 7, PICA = 15). When the risk factors of atherosclerosis were compared, patients with contrast enhancement of a distal VA on BB-enhanced MR imaging had the highest prevalence of hypertension (91.5% vs. 69.9%, respectively, *p* < 0.001). Additionally, the contrast enhancement of a unilateral VA on BB-enhanced MR imaging showed a significantly higher prevalence in patients with acute infarction on DWI (50.0% vs. 4.9%, *p* < 0.001). 

A comparison between positive DWI findings and negative DWI findings in this study is shown in Table 3. When the risk factors of atherosclerosis were compared, patients with positive DWI findings and a target territory of asymmetrical VA geometry had the highest prevalence of hypertension (92.2% vs. 72.6%, respectively, *p* = 0.003); diabetes (54.9% vs. 34.6%, respectively, *p* = 0.007); and smoking (41.2% vs. 24.1%, respectively, *p* = 0.013). Type 1 pattern (contrast-enhanced VA + no visualization of VA) showed a significantly higher correlation with acute infarction of target lesions (68.6% vs. 12.7%, *p* < 0.001).

## 4. Discussion

The present study focuses on the strong correlation between the contrast enhancement of a unilateral vertebral artery on BB-enhanced MR imaging finding and positive infarction findings in the DWI of the medulla, pons, or PICA territory. An acute infarction finding related to distal VA occlusion may be highly correlated to patients who show contrast-enhanced VA on BB-enhanced MR imaging, especially when there is no visualization of the VA on MR angiography findings.

Posterior circulation territories related to the vertebrobasilar arterial system include the brainstem, the thalami, the cerebellum, and parts of the occipital and temporal lobes. Though the VBA system supplies only 20% of cerebral blood flow, previous studies have shown that VBA occlusive events, both in the extracranial and intracranial portion, are critical points for understanding posterior circulation stroke [16,17]. Posterior circulation ischemia comprises 20–25% of all ischemic strokes [18]. Posterior circulation ischemia, in general, is a pathological condition of infarction within the VBA system, which occurs most commonly in the brainstem (48%) and the PICA territory (36%) and results from patients who have atherosclerosis with stenosis [19]. There are several common causes responsible for posterior circulation ischemia: embolism (40%), atherosclerosis in large vessels (3–35%), and cardiogenic embolism (24%) [20]. Anatomically, the vertebral artery is divided into 4 segments designated V1 to V4. The distal portion of the VA, the V4 segment, provides important penetrating branches to the medulla, anterior spinal communicators from each V4 region that join in the midline, and most notably the PICA [21]. Although the most common posterior circulation territory of an atherosclerotic occlusive event is within the V1 segment [22], the V4 segment and distal VBA junction are relatively common sites of atherosclerotic occlusion. 

Our study selectively includes 288 patients with asymmetrical VA geometry and acute neurological symptoms. Although, there have been only a few reports referring to unilateral abnormalities of VBAs in angiography (0.2% of cases in the population on cerebral angiography) [23], a higher rate of asymmetrical VA geometry is related to vertebrobasilar ischemic events [24]. Asymmetrical VA geometry can be classified into three types: aplasia, hypoplasia, or occlusion [25]. Distinguishing between the recent acute occlusion of a distal VA and the chronic occlusion or aplasia is difficult using only MR angiography due to a lack of visualization of the unilateral VA. Therefore, we used BB-enhanced MR imaging for the evaluation of acute occlusion of a unilateral distal VA in relation to acute infarction of the VA territory.

BB-enhanced MR imaging is a very informative tool for finding an intra-arterial thrombus in patients with acute infarction in the anterior and posterior circulation, and it is more accurate than an SVS on SWI because BB-enhanced MR imaging shows an occlusion site in patients with a thrombus or intracranial atherosclerosis [8,11]. In the case of no relation to contrast enhancement, if we compare BB-enhanced MR imaging to CT angiography, which is the current standard diagnostic tool for intracranial arterial occlusions, it shows an impressively similar diagnostic sensitivity and specification (100%, 99.8%, respectively) [26]. The main finding of BB-enhanced MR imaging in acute stroke patients is strong enhancement due to contrast stagnation in the front and rear of a thrombus or steno-occlusive lesion at the intracranial occlusion site [8,9,27]. Chung et al. [11] reported that the sensitivity of BB-enhanced MR imaging, with a strong enhancement for the detection of vessel occlusion in patients with posterior circulation stroke, was significantly higher compared to an SVS on SWI. We focused on the strong enhancement of an asymmetrical VA and acute infarction of the VA territory. 

In our study, patients with acute neurological symptoms were categorized into four types according to contrast enhancement in BB-enhanced MR imaging and the asymmetrical geometry of VA on MR angiography. Of the enrolled patients, 28.5% (82/288), including 65 without visualization of VA on MRA, showed contrast enhancement of an asymmetrical VA (type 1 = 65 and type 2 = 17). High-resolution BB MR imaging can be useful for patients with an asymmetrical VA, especially for the diagnosis of intracranial VA occlusion or luminal thrombosis [26,28]. The complete occlusion group and high-grade stenosis of an intracranial artery showed a concentric strong enhancement in BB-enhanced MR imaging [27]. Patients with an enhanced group in BB MR imaging and asymmetrical VA had a higher prevalence of atherosclerosis risk factors such as hypertension. Therefore, we thought that the contrast enhancement of an asymmetrical VA on BB-enhanced MR imaging occurring with the acute occlusion of the distal VA was due to an underlying atherosclerotic change or, less likely, a thrombotic embolism. An acute occlusion of the distal VA occurs when contrast stagnation occurs in the proximal portion of the occlusion site, and the stagnated contrast and slow flow might be not suppressed when using the BB MR technique. 

In our study, patients with enhanced VA and asymmetrical VA (50.0%; 41/82) had matched positive findings related to territorial VA (medulla = 19, pons = 7, PICA = 15). Additionally, the type 1 pattern (contrast-enhanced VA + no visualization of VA) showed a significantly higher correlation with acute infarction of target lesions. The blood supply of the posterior circulation occurs through the convergence of the VA, basilar artery, and PICA. In most cases, anatomically, the PICA is derived from the VA. If the PICA arises from an extradural site of C1 from the VA, it gives pial branches for the posterior surface of the medulla oblongata. If the PICA emerges in the intradural VA, it is responsible for the blood supply of the lateral surface of the brain stem and the ipsilateral cerebellum. Whether the emergence of the PICA happens intra- or extra-durally, the PICA segments of the distal VA play a key role in the vascularization of the brainstem [29]. The study has shown that PICA disease is usually associated with atherothrombosis and is correlated with the lateral–superficial area of the caudal medulla and/or dorsolateral portion of the rostral–middle medulla. Short-segment distal VA disease is mostly correlated with small lateral caudal and rostral–middle medullary lesions [30]. Posterior circulation stroke mainly occurs in a state of occlusion. However, it is also likely to be associated with the amount of reduction in blood flow through the VA due to decreased local perfusion in the proximal part of the vertebra–basilar system, which indicates the distal VA [31]. Therefore, as in our study, the occlusion of the distal VA, where the site is mostly the PICA, involves infarction of the medulla, pons, and PICA. If not, it still is responsible for the reduction of blood flow in the posterior circulation, which causes hypoperfusion of the brainstem region. In BB-enhanced MR imaging, contrast enhancement is present in acute VA occlusion or stenosis, and this finding is related to the asymmetrical VA geometry of MRA. Therefore, the contrast enhancement found in the VA on BB-enhanced MR imaging is a valuable method for diagnosing the cause of an acute stroke.

Our study has several major limitations and challenges. First, the small sample size of subtypes and its retrospective nature have the risk of potential selective bias. Larger cohorts and further studies would reinforce our study; Second, an imaging analysis was conducted only through consensus; Third, we did not perform an angiographic procedure due to focal or minor stroke; Finally, for a superior analysis, we would have preferred to classify distal VA geometry through different categories, such as hypoplastic, aplasia, and occlusion, using MRA to observe arterial outer contours and without being limited to no visualization of the distal VA in MRA [25]. Therefore, patients with chronic and total occlusion of VA are likely to be included in this study.

## 5. Conclusions

Strong enhancement of a unilateral distal VA on BB-enhanced MR imaging in patients with acute neurological symptoms was related to acute infarction of the medulla, pons, or PICA territory. Patients with an especially strong enhancement and no visualization of VA on MRA showed a higher prevalence of acute infarction. These findings in BB-enhanced MR imaging had a contrasting stagnation due to the acute occlusion of the distal VA and no suppression with the BB technique. Therefore, BB-enhanced MR imaging has a diagnostic value for the accurate detection of acute distal VA occlusion in patients with no visualization of a unilateral VA in MRA. 

## Figures and Tables

**Table 1:** Demographic of this study.

	N = 288
Age, mean and SD	71.6 ± 1.2
Male, *n* (%)	167 (58.0)
Hypertension, *n* (%)	219 (76.0)
Diabetes, *n* (%)	110 (38.2)
Smoking	78 (27.1)
Hyperlipidemia, *n* (%)	93 (32.3)
Previous stroke history, *n* (%)	97 (33.7)
Previous heart problem, *n* (%)	78 (27.1)
Pattern of contrast-enhanced VA, *n* (%)	
Type 1	65 (22.6)
Type 2	17 (5.9)
Type 3	129 (44.8)
Type 4	77 (26.7)
Contrast-enhanced VA (type 1 + 2), *n* (%)	82 (28.5)
Positive DWI, *n* (%)	51 (17.7)
Medulla	22
Pons	9
PICA	20

SD = standard deviation, VA = vertebral artery, DWI = diffusion-weighted imaging, PICA = posterior inferior cerebellar artery.

**Table 2:** Comparison of two groups with enhanced VA or non-enhanced VA on BB MR imaging.

	BB Enhanced Group (*n* =82)	BB Non-Enhanced Group (*n* = 206)	*p*
Mean age ± SD	73.1 ± 1.2	71.0 ± 0.9	0.216
Male, *n* (%)	56 (68.3)	111 (53.9)	0.025
Rt, *n* (%)	43 (52.4)	127 (62.0)	0.201
Hypertension, *n* (%)	71 (91.5)	144 (69.9)	<0.001
Diabetes, *n* (%)	35 (42.7)	75 (36.4)	0.323
Smoking, *n* (%)	25 (30.5)	53 (25.7)	0.412
Hyperlipidemia, *n* (%)	30 (36.6)	63 (30.6)	0.326
Previous stroke history, *n* (%)	28 (34.1)	69 (33.5)	0.916
Previous heart problem, *n* (%)	19 (23.2)	59 (28.6)	0.346
Positive DWI, *n* (%)	41 (50.0)	10 (4.9)	<0.001
Medulla	19	3	
Pons	7	2	
PICA	15	5	

BB = black blood, SD = standard deviation, VA = vertebral artery, DWI = diffusion-weighted imaging, PICA = posterior inferior cerebellar artery.

**Table 3:** Comparison between positive DWI findings and negative DWI findings in this study.

	Positive DWI (*n* = 51)	Negative DWI (*n* = 237)	*p*
Mean age ± SD	71.7 ± 1.6	71.5 ± 0.8	0.825
Male, *n* (%)	35 (68.6)	132 (55.7)	0.09
Rt, *n* (%)	43 (52.4)	127 (62.0)	0.247
Hypertension, *n* (%)	47 (92.2)	172 (72.6)	0.003
Diabetes, *n* (%)	28 (54.9)	82 (34.6)	0.007
Smoking, *n* (%)	21 (41.2)	57 (24.1)	0.013
Hyperlipidemia, *n* (%)	21 (41.2)	72 (30.4)	0.135
Previous stroke history, *n* (%)	16 (34.1)	81 (34.2)	0.701
Previous heart problem, *n* (%)	17 (33.3)	61 (25.7)	0.268
VA geometry pattern			<0.001
Type 1	35 (68.6)	30 (12.7)	
Type 2	6 (11.8)	11 (4.6)	
Type 3	8 (15.7)	122 (51.5)	
Type 4	2 (3.9)	74 (31.2)	

SD = standard deviation, VA = vertebral artery, DWI = diffusion-weighted imaging.

## Data Availability

The data presented in this study are available upon request from the corresponding author. The data are not publicly available due to privacy restrictions.
